# Microbiome’s role in musculoskeletal health through the gut-bone axis insights

**DOI:** 10.1080/19490976.2024.2410478

**Published:** 2024-10-10

**Authors:** Zhengrui Li, Qi Wang, Xufeng Huang, Yinteng Wu, Dan Shan

**Affiliations:** a School of Medicine, Shanghai Jiao Tong University, Shanghai, China; b Jiangsu University, Zhenjiang, China; c Faculty of Dentistry, University of Debrecen, Debrecen, Hungary; d Department of Orthopedic and Trauma Surgery, The First Affiliated Hospital, Guangxi Medical University, Nanning, China; e Faculty of Health and Medicine, Lancaster University, Lancaster, UK; f Department of Biobehavioral Sciences, Columbia University, New York, NY, USA

**Keywords:** Microbiome, musculoskeletal health, gut-bone axis, musculoskeletal disease, bone health

## Abstract

The interplay between the human microbiome and the musculoskeletal system represents a burgeoning field of research with profound implications for understanding and treating musculoskeletal disorders. This review articulates the pivotal role of the microbiome in modulating bone health, highlighting the gut-bone axis as a critical nexus for potential therapeutic intervention. Through a meticulous analysis of recent clinical research, we underscore the microbiome’s influence on osteoporosis, sarcopenia, osteoarthritis, and rheumatoid arthritis, delineating both the direct and indirect mechanisms by which microbiota could impact musculoskeletal integrity and function. Our investigation reveals novel insights into the microbiota’s contribution to bone density regulation, hormone production, immune modulation, and nutrient absorption, laying the groundwork for innovative microbiome-based strategies in musculoskeletal disease management. Significantly, we identify the challenges hindering the translation of research into clinical practice, including the limitations of current microbial sequencing techniques and the need for standardized methodologies in microbiome studies. Furthermore, we highlight promising directions for future research, particularly in the realm of personalized medicine, where the microbiome’s variability offers unique opportunities for tailored treatment approaches. This review sets a new agenda for leveraging gut microbiota in the diagnosis, prevention, and treatment of musculoskeletal conditions, marking a pivotal step toward integrating microbiome science into clinical musculoskeletal care.

## Influence of the human microbiome on osteoporosis and bone metabolism

The complex interplay between bone formation and resorption underlies bone metabolism,^1,2^ orchestrated by osteoblasts, osteocytes, and osteoclasts. After birth, bone mass accumulation ensues, peaking in adulthood before its gradual diminution. A direct correlation exists between peak bone mass and the propensity for osteoporosis development; a 10% increase in peak bone mineral density (BMD) can extend the onset of osteoporosis by approximately 13 years,^3,4^ while a 10% change in the age at menopause or the rate of non-menopausal bone loss is predicted to delay osteoporosis by approximately 2 years, underscoring the criticality of optimizing bone mass and strength early on. Osteoporosis, characterized by compromised bone microarchitecture, diminished bone mass, and an elevated fragility fracture risk, poses significant health challenges.^5^

**Figure 1.**
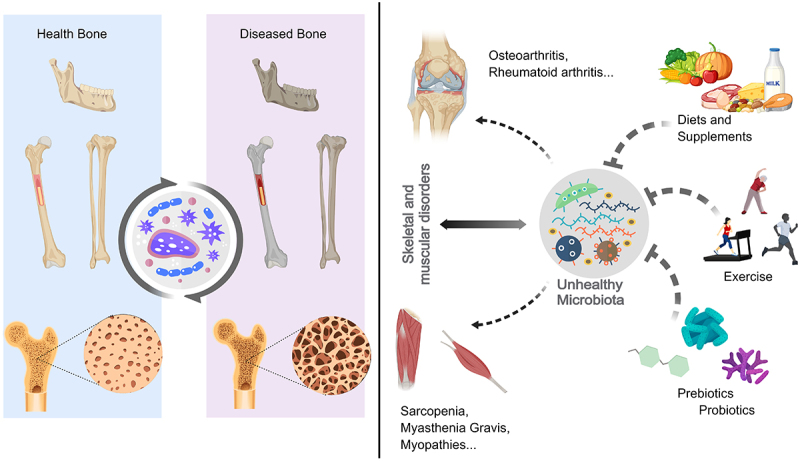
The influence of the human microbiome on the balance of the bone Homeostasis.

Concurrently, emerging evidence underscores the microbiome’s pivotal role in bone health.^6^ The microbiome interfaces with host metabolic processes, the immune system, and hormonal dynamics, playing indispensable roles in bone metabolism regulation (Figure 1).^7^ Osteoporosis risk is intricately linked to microbiome variations, which influence gut barrier function, calcium and vitamin D assimilation, immune system development, and hormone regulation, including estrogens and androgens.^8^ Dysbiosis, or microbial imbalance, may induce pro-inflammatory cytokine release, undermining gut barrier function and adversely affecting bone integrity. Research suggests potential benefits of probiotics and prebiotics in bone health enhancement or restoration.^9–11^ Probiotics are live microorganisms that, when administered in adequate amounts, confer health benefits on the host. Prebiotics are non-digestible food ingredients that selectively stimulate the growth and/or activity of beneficial microorganisms in the gut. Postbiotics are bioactive compounds produced by food-grade microorganisms during fermentation, which can have beneficial effects on the host. While these components may play a role in enhancing bone health by modulating the gut microbiota, the extent of their impact and the underlying mechanisms require further investigation. Moreover, the influence of the microbiota extends to joint and muscle health through immune modulation, energy metabolism, and fibrinogen synthesis. However, these broader implications of microbial health on musculoskeletal integrity should be interpreted with caution, as the current body of evidence is still evolving, and many studies are preliminary or observational in nature.

In summary, while the microbiome appears to play a role in bone health, it is essential to approach these findings with a critical perspective. More rigorous, large-scale studies are needed to establish causality, elucidate mechanisms, and determine the clinical significance of these associations. Understanding the limitations of current research will help guide future investigations and potential therapeutic applications.

## Nutrition and skeletal development

### Vitamin D and Bone mineral density

Evidence from randomized trials reveals that vitamin D supplementation in children with deficiency enhances BMD.^12,13^ The role of vitamin D extends beyond facilitating calcium absorption; it is instrumental in maintaining intestinal mucosal homeostasis, safeguarding epithelial barrier integrity, and enabling the transfer of microbial metabolites to the host.^14^ These processes are crucial for immune system maturation and modulating inflammatory responses. Notably, research involving adolescent girls has shown that high-dose vitamin D supplementation is associated with an increase in beneficial gut bacteria (both 112 taxa *p* < 0.05), such as *Firmicutes*, *Bifidobacteria*, and *Enterococci*.^15^ Additionally, there is evidence of a decrease in certain bacteria that can be potentially harmful in specific contexts, such as *Proteobacteria*. However, it is important to clarify that the role of *Lactobacilli* in gut health is complex and context-dependent. While some studies have identified situations where certain strains of *Lactobacilli* may be associated with negative health outcomes, these bacteria are generally considered beneficial and are widely used as probiotics (based on the International Scientific Association on Probiotics and Prebiotics (ISAPP) Guideline).

However, the studies often involve small sample sizes or specific populations, limiting generalizability. Additionally, while correlations exist, causality is not established, and the exact mechanisms remain unclear. The research involving Vitamin D Receptor (VDR) knockout (KO) mice further indicates an intricate relationship between vitamin D (VDRKO mice-Low BMD, from 7 and 24 weeks to 7–27 weeks age), its receptor, and the microbiome, but translating these animal model findings to human health outcomes requires further investigation.^16,17^

The interplay between vitamin D, the VDR gene, and the microbiome underscores the potential for microbiome-wide association studies (MWAS) in uncovering novel therapeutic avenues. Nevertheless, the ability of certain microbiota to modulate circulating vitamin D levels,^18^ as demonstrated by *Lactobacillus reuteri*‘s capacity to increase 25-hydroxyvitamin D significantly, and the capacity of bacteria like *Streptomyces griseolus* to activate vitamin D3, highlights the reciprocal influence between microbiota and vitamin D metabolism, which warrants more comprehensive human studies to confirm these preliminary findings.

In contrast to the robust data available for younger populations, the research on vitamin D’s effects on BMD in the elderly presents a nuanced picture. Several studies have shown a positive association between higher serum 25-hydroxyvitamin D levels and greater BMD in older adults. For instance, epidemiological evidence suggests that maintaining serum 25(OH)D levels around 75 nmol/L is beneficial for bone health in both young and old populations (P＜0.05).^19^ However, the relationship may not be entirely linear, and individual variability plays a significant role.

Furthermore, cross-sectional studies have demonstrated that serum vitamin D levels within the range of 30–90 nmol/L are positively correlated with hip BMD in the elderly (P＜0.05).^20^ However, some studies have found that while vitamin D supplements can significantly increase plasma vitamin D levels, the improvement in BMD is not significant (P.MBD＞0.05).^21^ Additionally, long-term studies have shown that the combined supplementation of calcium and vitamin D has a noticeable positive impact on hip bone density in elderly women, likely achieved by reducing bone resorption.^22^ However, the effectiveness of vitamin D supplements may vary depending on an individual’s baseline vitamin D levels, dosage, and duration of supplementation. In conclusion, while the importance of vitamin D in maintaining bone health is widely recognized, its specific impact on BMD in the elderly still requires further research and confirmation. Future studies should focus on large-scale, long-term randomized controlled trials to elucidate the effects of vitamin D supplementation on the bone health of various elderly populations, explore potential mechanisms, and determine the most effective supplementation strategies.

### Calcium: a pivotal mineral for bone density

Calcium’s significance in skeletal development, especially during periods of rapid growth, is well-documented.^23^ Emerging research suggests that short-chain fatty acids (SCFAs), produced by gut microbes, can enhance calcium absorption, thus improving bone density and strength.^24^ This finding holds promise for the use of prebiotics and postbiotics in addressing calcium deficiencies without the need to increase dietary calcium or supplementation directly.^25^

The bidirectional relationship exists between microbiota and calcium levels, where calcium supplementation can enrich microbial diversity,^26^ elevating populations of beneficial bacteria such as *Bifidobacteria*, *Ruminococcaceae*, and *Akkermansia*, is intriguing but also highlights the complexity of dietary interventions. In human studies, calcium and phosphorus supplementation have been linked to an increased presence of *Clostridium XVIII*, underscoring the complex interactions between dietary minerals and gut microbiota.^27^ This points to a need for more detailed mechanistic studies to fully understand these effects.

### Vitamin K and Bone health

Vitamin K plays a pivotal role in bone health, facilitating the conversion of osteoblasts to osteocytes, limiting osteoclast generation, and enabling the carboxylation of osteocalcin.^28–30^ Investigations into vitamin K have revealed its impact on the composition and structure of bone, with deficiencies in vitamin K, as produced by the microbiota, associated with weakened bone integrity.^31,32^ Among the gut microbiota, several bacterial species are known to produce vitamin K, specifically menaquinones (vitamin K2). These include various species of *Bacteroides*, *Escherichia coli*, *Lactococcus lactis*, and *Bacillus subtilis*.^33,34^ The presence and activity of these vitamin K-producing bacteria are crucial for maintaining adequate vitamin K levels, which in turn support bone health. Disruptions in the gut microbiome, such as those induced by antibiotic treatment, can significantly reduce the production of vitamin K, leading to compromised bone strength and mineral quality, as evidenced by reductions in tissue mechanical properties and bone mineral crystallinity.^35^

While the role of vitamin K-producing gut bacteria in bone health is evident, caution is warranted in interpreting these findings for clinical practice. For instance, individuals on vitamin K antagonists, such as warfarin, experience altered vitamin K metabolism, which can impact the gut microbiota and its production of menaquinones.^36,37^ This interaction may further complicate bone health, as the inhibition of vitamin K-dependent processes can lead to an increased risk of fractures and bone demineralization. Therefore, it is essential to consider these implications when prescribing vitamin K antagonists and to monitor bone health in these patients closely. Future research should aim to elucidate the specific mechanisms by which gut microbiota could influence vitamin K metabolism under various clinical conditions, including the use of vitamin K antagonists. This knowledge may inform strategies to mitigate adverse effects on bone health, potentially through dietary modifications, probiotic supplementation, or alternative therapeutic approaches.

### Dietary fiber and bone outcomes

The beneficial effects of dietary fiber on bone health, as evidenced by epidemiological studies, are partly attributed to the microbial fermentation of fiber into SCFAs (fiber intake-BMD β = 0.004, *p* = 0.038),^32,38,39^ such as propionate (C3) and butyrate (C4). These compounds have been shown to inhibit osteoclastogenesis and promote bone formation through various mechanisms,^40^ including the inhibition of histone deacetylase, enhanced osteoblast differentiation, and increased production of bone sialoprotein and osteoprotegerin.

Moreover, SCFAs can alter osteoclast metabolism, leading to reduced expression of osteoclast-related genes and a decrease in osteoclast differentiation.^41^ Animal studies have further demonstrated that diets enriched with specific oligosaccharides can significantly improve bone density,^42^ emphasizing the potential of dietary interventions targeting the microbiota for bone health improvement. However, it is important to note that dietary interventions rich in specific oligosaccharides (essentially sugars) might have significant implications for certain populations, such as individuals with diabetes. Therefore, such interventions should not be considered a one-size-fits-all solution.

The direct impact of dietary fiber on bone health in humans is not fully established, and human studies are needed to validate these findings and explore the optimal types and amounts of dietary fiber for bone health. Tailoring dietary recommendations to individual health conditions and metabolic needs will be crucial for maximizing the benefits of dietary fiber on bone outcomes.

## Hormonal influences on skeletal metabolism

Estrogen plays a critical role in maintaining bone homeostasis, especially after menopause.^43^ Recent studies have highlighted the microbiome as a significant regulator of estrogen levels. The microbial production of β-glucuronidase, an enzyme essential for estrogen activation, helps modulate estrogen levels.^44^ A decrease in microbial diversity can impair this process, leading to reduced circulating estrogen levels and contributing to the development of osteoporosis.^45^ Additionally, estrogen deficiency increases gut permeability and systemic inflammation, exacerbating bone loss. In a contrasting observation, research involving germ-free (GF) mice has demonstrated that these mice exhibit less bone loss following estrogen depletion. This phenomenon is attributed to the reduced production of osteoclastogenic cytokines, suggesting that microbial presence might influence the inflammation associated with bone degradation.^46^ While these findings offer promising directions for understanding the complex interactions between hormones and the microbiome, translating these results from animal models to human physiology demands caution. Differences in microbiome composition and hormonal regulation between species mean that direct applications and implications must be carefully considered.

Probiotic interventions, particularly those based on *Lactobacilli*, have shown promise in decreasing osteoclastogenic cytokines and boosting osteoprotegerin (OPG) expression, offering protection against ovariectomy-induced bone loss.^47,48^ Similarly, *Bifidobacterium longum* supplementation has similarly been demonstrated to mitigate bone loss in OVX rats, highlighting the therapeutic potential of microbiota modulation in combating osteoporosis.^45^ Moreover, preventing gut leakage with antibiotics or *Lactobacillus* reuteri supplementation has proven effective against glucocorticoid-induced osteoporosis in mice.^49–51^ The microbiome’s role in regulating dihydrotestosterone (DHT) levels in the distal gut also underscores the necessity for further research to elucidate the broader implications of microbiota in hormone metabolism and bone health.

## The microbiome-bone immuno-inflammation axis

**Figure 2.**
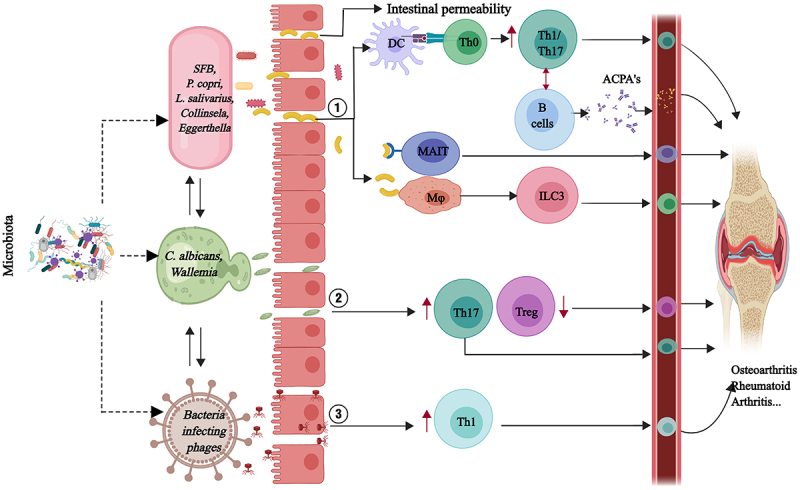
The microbiome and bone immuno-inflammation. This figure illustrates the complex interplay between gut microbiota, intestinal barrier integrity, and the immune system in the pathogenesis of osteoarthritis and rheumatoid arthritis. Specific gut microbiota species (*SFB, P. copri, L. salivarius, Collinsella, Eggerthella*) disrupt the intestinal epithelial barrier, increasing permeability and facilitating the translocation of microbial antigens and metabolites into the systemic circulation. This triggers immune activation, with dendritic cells (DC) activating naïve T cells (Th0) into Th1 and Th17 cells, and B cells producing anti-citrullinated protein antibodies (ACPAs). Additional immune cells, such as macrophages (Mφ), mucosal-associated invariant T cells (MAIT), and type 3 innate lymphoid cells (ILC3), modulate the inflammatory response. Fungal species (*Candida albicans, Wallemia*) and bacteriophages also contribute to barrier disruption and Th1 responses. The systemic dissemination of pro-inflammatory mediators, including Th1/Th17 cytokines and ACPAs, leads to joint inflammation, chronic inflammation, autoimmunity, and the joint damage characteristic of osteoarthritis and rheumatoid arthritis.

The human microbiome, with its intricate network of microorganisms, is increasingly recognized as a key modulator of immune system development and function.^52^ Specifically, the gut microbiota is known to initiate pathogenic immune responses through the activation of pattern recognition receptors on immune cells, such as dendritic cells and macrophages, which reside in the mucosal layers of the gastrointestinal tract (Figure 2). These antigen-presenting cells (APCs) capture microbial antigens and travel to the regional lymph nodes, where they present these antigens to naïve T cells, leading to their activation and differentiation into various effector T cell subsets.

The trafficking of disease-mediating immune cells to the site of disease induction is a highly regulated process involving a complex interplay of chemokines, adhesion molecules, and other signaling pathways. Once activated, these T cells migrate through the bloodstream and are guided by chemokine gradients to the target tissue, where they contribute to the pathogenesis of diseases such as osteoporosis. In the context of bone health, the immune cells can influence osteoclastogenesis and bone resorption, leading to the characteristic loss of bone mass observed in osteoporosis.

In addition, the microbiome plays a pivotal role in the maturation of the immune system,^53^ significantly affecting cytokine production and the development of lymphocytes, particularly T-helper cells. As individuals age, especially under estrogen-depleted conditions, there is a marked increase in the production of inflammatory and osteoclastogenic cytokines by T cells, notably TNF-α and RANKL.^54–56^ These cytokines are critical in bone resorption processes, directly impacting bone health. The microbiota’s capacity to modulate the levels of these cytokines, and consequently affect bone density and health, is mediated through the activation of NOD-like receptors 1 and 2 (NOD1 and NOD2).^57–59^ NOD1 and NOD2 are intracellular pattern recognition receptors that can identify molecular patterns derived from microbes and initiate downstream signaling pathways. Within T cells, the activation of NOD receptors triggers the activation of nuclear factor kappa B (NF-κB) and other transcription factors. These factors then translocate to the nucleus and bind to the promoter regions of the TNF-α and RANKL genes, facilitating the transcription and translation of these genes. This process leads to the release of increased levels of TNF-α and RANKL from T cells, exacerbating bone resorption and inflammatory processes. Consequently, the microbiota, through the modulation of NOD receptor activity, can significantly influence bone health via the metabolic products and cytokines it produces.

The influence of the microbiota on bone metabolism extends to the delicate balance between osteoclast and osteoblast activity, which is critical for maintaining bone homeostasis. One mechanism by which the microbiota may impact this balance involves Toll-like receptor 5 (TLR5). TLR5, predominantly expressed on immune cells such as dendritic cells and macrophages, can modulate immune responses and has been implicated in bone homeostasis.^60–63^ Although TLR5 is not directly expressed on osteoclasts, its activation on immune cells can indirectly affect osteoclastogenesis by influencing the secretion of cytokines that regulate bone resorption and formation.

In TLR5-deficient mice (TLR5-KO), an increase in periosteal bone formation has been observed, which can be mitigated by altering the microbiome.^64^ This finding suggests that the microbiota may modulate bone structure and health through indirect mechanisms involving TLR5 signaling in immune cells. Furthermore, it is becoming increasingly evident that the microbiota can also be influenced by lifestyle factors such as physical activity.^65^ Studies have shown that increased physical activity in mice is associated with an increased abundance of *Bifidobacteriaceae*, a bacterial family associated with reduced intestinal inflammation and improved bone density.^66^ This association further illustrates the multifaceted impact of lifestyle factors on bone health, mediated through changes in the microbiome.

An important aspect that warrants further investigation is whether the disruption of the epithelial barrier of the gut is a common feature in all bone diseases. Current evidence suggests that increased intestinal permeability, often referred to as “leaky gut,” may play a role in the pathogenesis of several bone diseases.^67^ The disruption of the gut barrier allows for the translocation of microbial products and toxins, which can trigger systemic inflammation and immune responses that negatively impact bone health.^68–70^ However, it is crucial to determine if this mechanism is universal across different bone diseases or if it is more specific to certain conditions. Further research is needed to elucidate the relationship between gut barrier integrity and bone health, and to identify potential therapeutic targets that can restore barrier function and mitigate bone disease progression. Understanding the role of gut barrier disruption in bone diseases could lead to novel interventions aimed at maintaining or restoring gut integrity as a means of preserving bone health.

## Current clinical research status of microbiota - osteoporosis interactions

The complex ecosystem of the human microbiome plays a pivotal role in numerous aspects of health and disease, including its emerging influence on bone health and osteoporosis. Recent advances in clinical research have begun to unravel the intricate connections between microbiota compositions and their impact on osteoporosis, a condition characterized by reduced bone mass and increased fracture risk. This burgeoning field of study opens new avenues for understanding the pathophysiology of osteoporosis and potentially heralds novel microbiome-based therapeutic strategies. The following sections delve into the current clinical research status of microbiota-osteoporosis interactions, encompassing correlational studies, microbiome-based clinical trials, and the microbiome’s effects on skeletal muscle mass, function, and joint health, as well as its interplay with immune responses. Through a comprehensive examination of these areas, we aim to illuminate the multifaceted relationships between the microbiome and bone health, highlighting both established findings and avenues for future research.

### Correlational studies: insights and implications

Recent research has shed light on the microbiome’s composition in individuals with osteoporosis,^71,72^ revealing a higher prevalence of *Actinobacteria, Eggerthella, Clostridium Cluster XIVa*, and *Lactobacillaceae* (P＜0.01).Conversely, this group also showed diminished levels of *Escherichia/Shigella* and *Veillonellaceae* (P＜0.05). These findings, while intriguing, did not demonstrate significant microbial diversity differences between the groups. Another study unveiled an increased microbial diversity and abundance of *Dialister* and *Faecalibacterium* in individuals with osteoporosis as opposed to those with normal BMD (FDR-corrected *p* < 0.05).^73^ Extensive studies from China correlating the presence of specific microbiota such as *Bifidobacterium, Roseburia, Lactobacillus*, and others with bone density underscore the complexity of these associations (*p* < 0.001).^74^

These diverse outcomes highlight the critical need for substantial sample sizes and rigorous control measures in research to elucidate potential microbiota-osteoporosis links.^75^ It is important to note that results from non-Asian studies sometimes present contradictory findings, underscoring the influence of regional and cultural differences, such as diet, on gut microbiota composition. For instance, studies conducted in North America and Europe have shown significant differences in the relationship between microbiome composition and bone density, suggesting that environmental and dietary factors play a crucial role,^76,77^ whereas no bone loss was observed in the Lactobacillus-treated group (−0·01%, −0·50 to 0·48, P＜0.05).

Many of the included studies have accounted for confounding variables like diet, given its substantial variation across cultures and nations, which can significantly affect gut microbiota composition. These diverse outcomes highlight the critical need for large sample sizes and rigorous control measures in research to elucidate potential links between microbiota and osteoporosis. Most studies mentioned rely on sequencing results, focusing on relative changes and abundance comparisons of microbial communities. While these sequencing data reveal differences in microbial community composition, they often do not provide direct correlation coefficients or levels of significance. Therefore, interpreting these findings requires further statistical analyses and large-scale studies to validate these associations and determine their specific correlation strength and significance.

### Microbiome-based clinical trials: pioneering treatments

In Sweden, groundbreaking clinical trials have indicated that probiotics may play a significant role in mitigating bone loss in postmenopausal women.^78^ One trial reported a reduction in tibial volumetric BMD loss following daily supplementation with *Lactobacillus reuteri 6475* over one year.^79^ Another study documented decreased lumbar spine BMD loss in participants after a 12-month regimen of a specific probiotic blend compared to a placebo group.^80^ Meanwhile, a Japanese clinical trial observed notable improvements in hip bone density after 24 weeks of *Bacillus subtilis* C-3102 supplementation, although no significant changes were reported in lumbar spine BMD.^81^ Additionally, an Iranian study highlighted the potential of GeriLact, a multi-strain probiotic, in lowering serum CTX levels in postmenopausal women, suggesting a decrease in bone turnover.^82^

### Microbiome’s influence on skeletal muscle mass and function

Explorations into the gut-bone axis have unveiled its potential implications in the pathogenesis of muscle wasting diseases.^83,84^ This relationship appears to influence muscle anabolism, which is regulated by factors such as diet, inflammation, and insulin sensitivity.^85–87^ Experimental transplants of microbiota into germ-free mice have resulted in increased muscle mass and a reduction in atrophy markers.^88^ Moreover, interventions with short-chain fatty acids have been shown to ameliorate muscle detriments,^89^ underscoring the microbiome’s role in muscle health.

In addition to these findings, recent studies have highlighted significant changes in the gut microbiota composition in patients with viral infections, which can also be relevant to muscle mass and function. For example, the gut microbiota of patients with HIV has shown notable alterations that correlate with muscle wasting conditions (*p* < 0.001).^90^ Similarly, influenza and COVID-19 infections have been linked to changes in gut microbiota that might impact muscle recovery and overall function.^91,92^

Furthermore, research on elderly populations has demonstrated that the composition of the gut microbiome changes with age, potentially influencing muscle mass and strength. Older adults often experience sarcopenia, a condition characterized by loss of muscle mass and function, which has been associated with an altered gut microbiome.^93^ Studies suggest that probiotics and prebiotics can beneficially modulate the gut microbiota in older adults, potentially mitigating sarcopenia.^94,95^

These findings collectively underscore the importance of the gut-muscle axis and suggest that targeting the microbiome could be a viable strategy to preserve or enhance muscle mass and function, especially in clinical populations and the aging demographic.

### Microbiome impacts on joint health

Osteoarthritis (OA), characterized by degenerative joint disease often linked with obesity and inflammation, has been associated with microbiome compositions.^96–98^ Interventions using *Lactobacillus paracasei* or prebiotics like oligofructose have demonstrated potential in mitigating obesity-mediated OA risks.^99–102^ Notably, elevated bacterial lipopolysaccharides (LPS) levels in serum and synovial fluid have been correlated with exacerbated OA pathology.^103,104^ Furthermore, the abundance of *Streptococcus species* has been associated with increased knee pain and inflammation, while specific probiotics have shown promise in slowing knee OA progression.^105^

### Microbiome and immune response interactions

In rheumatoid arthritis (RA) pathogenesis, multiple subsets of immune cells play crucial roles. T cell subsets, particularly Th17 and Th1, contribute significantly to inflammation through the secretion of pro-inflammatory cytokines such as IL-17 and IFN-γ.^106,107^ Regulatory T cells (Tregs) regulate immune responses negatively by secreting anti-inflammatory cytokines like IL-10 and TGF-β; however, in RA patients, the quantity or function of Treg cells may be impaired.^108,109^ B cells not only produce antibodies but also activate T cells and primary fibroblast-like synoviocytes (FLS) as antigen-presenting cells and participate in immune regulation by secreting cytokine.^110,111^ Macrophages have dual roles in synovial inflammation in RA, clearing debris and dead cells through phagocytosis while exacerbating inflammation by secreting pro-inflammatory cytokines like TNF-α and IL-6.^112^ Dendritic cells, crucial in activating T cells and initiating immune responses, may promote autoimmune reactions in RA through abnormal antigen presentation processes.^113^ Neutrophils participate in early inflammation in RA by releasing neutrophil extracellular traps (NETs) and other inflammatory mediators.^114–116^ Mucosal-associated invariant T cells (MAIT cells), enriched in synovial fluid of RA patients, may contribute to RA pathogenesis through their influence on metabolites and cytokine production.^117^

In addition, changes in the microbial composition of RA are also a key factor, especially the interaction between intestinal microbiota and immune cells plays a crucial role in the occurrence and development mechanism of RA. The oral and gut microbiome compositions are altered in RA, with enrichments in *Prevotella* and *Lactobacillus* species noted in RA cases.^118,119^ Additionally, *Cryptobacterium curtum*, known for its association with citrulline production, has been found in higher concentrations in RA individuals.^120^ In particular, butyrate producing bacteria in the gut microbiota such as *Fusobacterium* XIVa Group and *Lachnospiraceae* have been associated with intestinal microbial imbalance in RA patients. These microbiota limit the development of autoimmune responses by promoting the differentiation of follicular regulatory T cells (TFR), especially in experimental models of arthritis showing potential to inhibit arthritis symptoms.^121^ Secondly, *Faecalibacterium prausnitzii* (*F. prausnitzii*) has shown significant anti-inflammatory effect on *Prausnitzii* by regulating IL-17-producing cells, changing the concentration of SCFAs and microbiome composition in experimental RA mouse models.^122^ Finally, the *Eggerthella lenta* study revealed a link between it and the pathogenesis of RA, in particular that it exacerbates the development of arthritis by regulating the production of CXCL5 and CD4+ T cells, pro-inflammatory factors IL-17 and IFN-γ, as well as affecting the dysregulation of the gut microbiota.^123^ It may be possible to regulate the pathogenesis of RA by regulating specific microorganisms to affect immune cells and their subpopulations in the future.

Probiotic interventions, such as *Lactobacillus casei* have indicated its potential in inhibiting RA induction and safeguarding against bone damage in rat models,^124^ highlighting the microbiome’s role in modulating immune responses and its implications, and perhaps probiotic interventions could be an important target for RA management.^120,125,126^

## Limitations

Despite significant progress in research on the relationship between the gut microbiome and bone health, several limitations persist. First, many studies are primarily based on animal models or small-scale human studies, which may not fully reflect the complex biological processes in humans. Therefore, large-scale, long-term population studies are needed to validate these findings and determine their applicability across different populations.^127^

Secondly, there are methodological differences among existing studies, including variations in study design, sample size, intervention methods, and data analysis techniques, making it challenging to compare and synthesize results from various studies. Additionally, the diversity and individual differences in the gut microbiome present another major challenge. Each person’s gut microbiome is influenced by various factors such as diet, lifestyle, medication use, and genetic background, all of which need to be controlled for in study designs.^128^

Thirdly, although studies have shown the potential of probiotics and prebiotics in improving bone health, the specific mechanisms remain unclear. The effects of different probiotic strains and prebiotics may vary, and in some cases, they might even have opposite effects, necessitating more precise and personalized research. Current studies often fail to adequately consider potential confounding factors such as diet, exercise, medication use, and disease status, which can significantly impact study results and should be accounted for in research design and data analysis.^129^

Furthermore, current research mainly focuses on specific microbial groups and metabolites, overlooking the complexity of the overall gut microbiome ecosystem. Future research should employ systems biology and multi-omics approaches to comprehensively reveal the multidimensional interactions between the gut microbiome and bone health, providing a more complete scientific basis for developing new prevention and treatment strategies.^130^

Finally, the gut-bone axis is likely bidirectional, where changes in bone health can influence the gut microbiota, and vice versa.^131^ For example, bone-derived hormones like osteocalcin have been shown to affect glucose metabolism and gut microbiota composition.^132,133^ This interplay highlights the need for a holistic approach in studying the microbiome, considering both intestinal and extra-intestinal factors that might contribute to gut dysbiosis and subsequent bone health issues.

In conclusion, while significant strides have been made in understanding the gut microbiome’s role in bone health, addressing these limitations through comprehensive, well-designed studies and considering the bidirectional nature of the gut-bone axis will provide deeper insights and more effective therapeutic strategies.

## Research frontier and challenges

In the evolving landscape of musculoskeletal research, the human microbiome has emerged as a critical frontier, providing hopeful insights and opening new therapeutic pathways. The exploration of the microbiome’s impact on bone health and osteoporosis marks a pivotal shift toward integrating microbial dynamics with traditional musculoskeletal research paradigms. However, effectively navigating this complex field requires overcoming several challenges.

First and foremost, the current reliance on 16S rRNA sequencing methods, while practical, falls short in accurately identifying specific bacterial strains and elucidating their functional contributions.^134,135^ The advent of metagenomic sequencing offers a powerful solution to these limitations, enabling a more precise characterization of microbial communities and their roles in health and disease.^136,137^

Another significant challenge is the common use of small cohort sizes in microbiome research, which compromises the applicability of findings across broader populations. Expanding study populations to encompass a wider range of demographic and genetic backgrounds is crucial for validating and deepening our understanding of the microbiome’s effects on bone health. Additionally, the field suffers from a lack of standardized methodologies across the entire spectrum of microbiome research, from sample collection and processing to data analysis. Establishing uniform protocols is essential for improving the comparability and reproducibility of research outcomes, thereby facilitating scientific progress.

Despite these obstacles, the article highlights the encouraging results from clinical trials that emphasize the microbiome’s integral role in bone metabolism. These findings not only bring us closer to unraveling the complex relationship between the microbiome and bone health but also lay the groundwork for innovative therapeutic strategies. As research progresses, the potential for microbiome-centered approaches to musculoskeletal disease management expands, heralding a new era of personalized medicine. The deepening exploration of our microbiota and its impact on our health foreshadows a future where treatments are as unique as the microbial signatures they aim to modulate, transforming the treatment landscape for musculoskeletal diseases.

## Abbreviations

BMD: Bone Mineral Density
VDR,: Vitamin D Receptor
MWAS: Microbiome-Wide Association Studies
SCFAs: Short Chain Fatty Acids
OPG: Osteoprotegerin
DHT: Dihydrotestosterone
NOD1: NOD-like receptors 1
NOD2: NOD-like receptors 2
OA: Osteoarthritis
RA: Rheumatoid Arthritis
